# Computed tomography in adult patients with primary ciliary dyskinesia: Typical imaging findings

**DOI:** 10.1371/journal.pone.0191457

**Published:** 2018-02-06

**Authors:** Sabine Dettmer, Felix Ringshausen, Jens Vogel-Claussen, Jan Fuge, Amir Faschkami, Hoen-oh Shin, Nicolaus Schwerk, Tobias Welte, Frank Wacker, Jessica Rademacher

**Affiliations:** 1 Department of Diagnostic and Interventional Radiology, Hannover Medical School, Hannover, Germany; 2 Department of Respiratory Medicine, Hannover Medical School, Hannover, Germany; 3 Biomedical Research in Endstage and Obstructive Lung Disease Hannover (BREATH), German Center for Lung Research (DZL), Hannover, Germany; 4 Department of Pediatric Pneumology and Neonatology, Hannover Medical School, Hannover, Germany; Telethon Institute for Child Health Research, AUSTRALIA

## Abstract

**Objectives:**

Among patients with non-cystic fibrosis bronchiectasis, 1–18% have an underlying diagnosis of primary ciliary dyskinesia (PCD) and it is suspected that there is under-recognition of this disease. Our intention was to evaluate the specific features of PCD seen on computed tomography (CT) in the cohort of bronchiectasis in order to facilitate the diagnosis.

**Materials and methods:**

One hundred and twenty-one CTs performed in patients with bronchiectasis were scored for the involvement, type, and lobar distribution of bronchiectasis, bronchial dilatation, and bronchial wall thickening. Later, associated findings such as mucus plugging, tree in bud, consolidations, ground glass opacities, interlobular thickening, intralobular lines, situs inversus, emphysema, mosaic attenuation, and atelectasis were registered. Patients with PCD (n = 46) were compared to patients with other underlying diseases (n = 75).

**Results:**

In patients with PCD, the extent and severity of the bronchiectasis and bronchial wall thickness were significantly lower in the upper lung lobes (p<0.001-p = 0.011). The lobar distribution differed significantly with a predominance in the middle and lower lobes in patients with PCD (<0.001). Significantly more common in patients with PCD were mucous plugging (p = 0.001), tree in bud (p <0.001), atelectasis (p = 0.009), and a history of resection of a middle or lower lobe (p = 0.047). Less common were emphysematous (p = 0.003) and fibrotic (p<0.001) changes. A situs inversus (Kartagener’s Syndrome) was only seen in patients with PCD (17%, p <0.001).

**Conclusion:**

Typical imaging features in PCD include a predominance of bronchiectasis in the middle and lower lobes, severe tree in bud pattern, mucous plugging, and atelectasis. These findings may help practitioners to identify patients with bronchiectasis in whom further work-up for PCD is called for.

## Introduction

Bronchiectasis is a condition which has permanent dilation of the bronchi and bronchioles as a result of the destruction of the bronchial wall and elastic connective tissue. The condition commonly starts with narrowing of the bronchial tree triggered by an infection which may lead to inflammation and injury of the epithelium if it becomes chronic [1]. There are various aetiologies which may result in bronchiectasis, including postinfective, chronic obstructive pulmonary disease, connective tissue disease, immunodeficiency, and inherited disorders such as primary ciliary dyskinesia (PCD) [2].

PCD is an autosomal, recessive, inherited disorder resulting in ultrastructural defects of the ciliary apparatus with consequent abnormal or absent beating of cilia [3]. This interferes with normal mucociliary clearance and causes repeated respiratory infections leading to airway damage and increasing the risk for the development of bronchiectasis [4]. Among patients with non-cystic fibrosis bronchiectasis, 1–18% have an underlying diagnosis of inherited disorders including PCD, and it is suspected that there is insufficient recognition of this disease [5–7].

### Diagnosis of PCD

The diagnosis of PCD is complex and is based on suggestive clinical findings, e.g. a chronic wet cough and chronic rhinitis since infancy, a pathologic high-frequency video microscopy analysis (HVMA), an ultrastructural ciliary defect seen in transmission electronic microscopy (TEM) and/or immunofluorescence microscopy (IF), and/or nasal NO measurement and/or the detection by genotyping of a biallelic disease-causing mutation [8]. Results from these different tests are often inconclusive thus making the diagnosis difficult. The full diagnostic work-up requires a specific technical set-up available in only very few medical centers. Knowledge of PCD as the underlying condition of bronchiectasis is of particular importance due to the possibility of upcoming, targeted therapies such as inhaled inhibitors of the epithelial sodium channel (ENaC), the emphasis of physiotherapy, rehabilitation, and consequent treatment of upper airway complications. Furthermore, the awareness of PCD is still low. This might be a reason for an inacceptable delay of a specialized diagnostic workup and sufficient treatment which increases the risk of irreversible lung destruction [9].

### Imaging in bronchiectasis

Imaging is of crucial importance in diagnosing and monitoring bronchiectasis. After the first description of bronchiectasis in CT in 1982 [10], technical advances increasingly improved the sensitivity. Bronchography was replaced by CT as the gold standard for diagnosing bronchiectasis [11]. Currently, CT is widely used in adults for diagnosing, characterization, and monitoring of bronchiectasis. Further on CT is obligatory for inclusion in national and international bronchiectasis registries [12,13]. Morphological criteria seen on CT include bronchial dilatation with respect to the accompanying pulmonary artery, a lack of tapering, and identification of bronchi within 1 cm of the pleural surface [14]. The broncho-arterial ratio provides evidence regarding the severity of the bronchiectasis, and a ratio >1 is considered as abnormal [15]. Depending on its appearance, bronchiectasis is classified as cylindrical (uniform dilatation with non-tapering walls), varicose (undulating dilatation) or cystic [16]. In addition to its appearance, the distribution of bronchiectasis can also help to characterize the disease. There are several scores which had been implemented for evaluation of bronchiectasis; well-known are the scores of Bhalla et al. [17] and the modified score of Reiff et al. [18]. Scores have been shown to correlate with the extent of physiological impairment in lung function tests [19,20] and are used to estimate the disease severity and for monitoring the disease in patients with bronchiectasis [20]. Nevertheless, several studies showed poor results regarding revealing the disease etiology, and the correct diagnosis of the underlying disease could be made on CT in less than 2/3 of patients [18,21,22]. In patients with PCD, CT typically shows varicose bronchiectasis, a chronic volume loss, a tree in bud pattern, and mucous plugging [4]. Later, a predominance of bronchiectasis in the middle and lower lung lobes is typical of PCD [23] and a lobectomy has often been performed when there is evidence of PCD [9].

### Purpose

The purpose of this study is to further evaluate specific findings of PCD on CT in order to determine patterns that differ from those of patients with bronchiectasis due to other underlying diseases. This might be of help to practitioners in identifying patients who require a further diagnostic workup for PCD.

## Materials and methods

### Study design

This study has been approved by the Internal Review Board (No. 2675–2015) of our institution (Hannover Medical School). The patient data and CT were evaluated in a pseudonymous form. IRB waived the requirement for informed consent. This is a retrospective, single center study.

We included all of the patients between the ages of 18 and 75 years who visited our specialized Outpatient Department between 2011 and 2017 due to their bronchiectasis and who underwent a CT examination in our Radiology Department during this time. Patients with definite or probable PCD, according to Werner et al. [24], were also included when they had an CT examination at another medical institution of sufficient quality. All patients had undergone an extensive work-up in accordance with the BTS guidelines in order to determine the cause of their bronchiectasis [25]. Individual patients were classified as having a definite, probable, possible or no PCD diagnosis, similar to that previously described [24]. Patients who fulfilled the following diagnostic criteria had a diagnosis of definite PCD: 1) clinical presentation consistent with PCD and 2) consistent findings specific for PCD according to at least two methods, i.e. high-frequency video microscopy analysis (HVMA), transmission electronic microscopy (TEM), immunofluorescence microscopy (IF), nNO (<200 ppb or 77nL/min) or biallelic disease-causing mutations determined by genotyping. All individuals with typical clinical symptoms and one abnormal diagnostic test were considered to have probable PCD.

### CT data acquisition

The CT examinations were performed in clinical stability and no CT during acute exacerbation was evaluated. Most of the CT examinations were performed in our Radiology Department. CT examinations were obtained with a 64 row MDCT (Lightspeed VCT, GE Healthcare, Milwaukee, WI, USA) or a 16 row MDCT (Lightspeed 16, GE Healthcare, Milwaukee, WI, USA). All CT data were acquired volumetrically using a standard dose protocol with 120 kV and 100 mAs. CT data were reconstructed with a slice collimation of 1.25 mm and an interval of 1 mm. Intravenous contrast medium was used if it was required for the particular clinical situation. Thirty-seven CT examinations were performed externally in patients with PCD using differing protocols and a slice thickness varying from 1.25 mm to 5 mm. CT with insufficient quality due to a slice thickness >5mm or to severe motion artifacts were excluded.

### CT features and semiquantitative scoring

All CT examinations were evaluated by a radiologist blinded to the diagnosis with eight years of clinical experience reading chest CTs (SD). Bronchiectasis was diagnosed according to the criteria described by Naidich [10]. The Reiff-score [18] was used for evaluation of bronchiectasis. Therefore, each lobe (with the lingula considered as a separate lobe) was scored for the extent of involvement (0 = none, 1 = one or partial segment, 2 = two or more segments); severity of bronchial dilatation (0 = normal, 1 = less than twice the diameter, 2 = 2–3 times the diameter, and 3 = more than 3x the diameter of the adjacent pulmonary artery); severity of the bronchial wall thickening (0 = normal, 1 = half the diameter, 2 = 0.5-1x diameter, and 3 = more than 1x the diameter of the adjacent pulmonary artery); type of bronchiectasis (1 = cylindrical, 2 = varicose, or 3 = cystic according to Reid [16]). Later, the lobar distribution of bronchiectasis (0 = widespread, 1 = predominantly upper lobe, 2 = predominantly middle lobe, 3 = predominantly lower lobe, 4 = middle and lower lobes equally involved, or 5 = unclassifiable) was registered [18]. In case of situs inversus or heterotaxy, right-sided changes were assigned to the left site according to the architecture of the lobes.

In addition to the Reiff-score, collateral findings were registered. Therefore, mucous plugging, tree in bud, peripheral and central consolidations, peripheral and central ground glass opacities, interlobular septal thickening and intralobular lines were scored (0 = none, 1 = 1–3 bronchopulmonary segments involved, 2 = >3 bronchopulmonary segments involved) for the whole lung. Mosaic attenuation, atelectasis, emphysema and situs inversus / heterotaxy were classified as present / absent. It was subsequently indicated if bronchiectasis was predominant in the middle and lower lobes and if both mucous plugging and tree in bud were present in more than three segments. All terms were used according to the definition of the Fleischner Society [14]. Last it was registered if there was a (sub)total atelectasis or a condition after resection of a lower or middle lobe / lingula.

### Statistics

The IBM SPSS Statistics (version 24.0, IBM Corp., Armonk, NY, USA) statistical software program was used to analyse the data.

Categorical variables are shown as numbers (n) and percentages (%). Ordinal scaled variables are shown as mean ±SD or median with interquartile range unless indicated otherwise. Imaging features were compared between patients with and without PCD. Group comparisons were performed using the Mann-Whitney-U-test for ordinal scaled and the Chi-square-test for categorical variables. All reported p-values are two-sided unless indicated otherwise; p-values <0.05 were considered statistically significant.

## Results

### Patient characteristics and diagnostic work-up

One hundred and twenty-one patients with bronchiectasis were included in our study, 46 of them with PCD as the underlying disease. The patient characteristics are shown in Table 1. The performed PCD investigations showed nasal nitric oxide in 46 patients, HVMA in 33 patients, TEM in 28 patients, IF in four patients, and genetic analysis in 16 patients. In summary, 29 patients had a definite, 17 patients a probable, and no patient had a possible diagnosis of PCD. Details including the results of the diagnostic work-up are shown below in Table 2.

**Table 1:** Patient characteristics.

Characteristics		Non-PCD (n = 75)	PCD (n = 46)
**Sex—n (%)**	Male	32 (43)	15 (33)
	Female	43 (57)	31 (67)
**Age when CT performed–Median (Range)**		49 (18–75)	38 (18–72)
**Age at diagnosis–Median (IQR)**		49 (25–58)	40 (31–50)
**Cause–n (%)**	Idiopathic	25 (33)	
	Asthma/ ABPA	11 (11)	
	Immunodeficiency	10 (13)	
	COPD/ A1AT	6 (8)	
	Postinfectious	6 (8)	
	GvHD	6 (8)	
	NTM lung disease	3 (4)	
	CFTR- related disorder	3 (4)	
	Connective tissue disease	3 (4)	
	Other	2 (3)	
**Smoker—n (%)**	Current	4 (5)	2 (4)
	Former	20 (26)	6 (13)
	Never	52 (68)	39 (83)
**Microbiology- n (%)**	Pseudomonas aeruginosa	15 (20)	18 (38)
	Staphylococcus aureus	7 (9)	4 (9)
	Aspergillus fumigatus	7 (9)	2 (4)
	E. coli	1 (1)	3 (6)
	Serratia marcescens	3 (4)	2 (4)
	Streptococcus pneumonia	3 (4)	0 (0)
	Haemophilus influenza	3 (4)	4 (9)
	No pathogen	33 (43)	14 (30)
**Lung function–Mean (SD)**	FEV1 percent predicted	66 (+-27)	70(+-23)
	FVC percent predicted	80 (+-26)	85 (+-23)
**Body mass Index–Mean (SD)**		25 (+-6,2)	23 (+-3,1)
**BSI-Score–n (%)**	- 0–4	19 (25)	15 (32)
	- 5–8	15 (20)	17 (36)
	- >9	42 (55)	15 (32)
**Exacerbations–Median (IQR)**		2 (0–4)	2 (0–3)
**Hospitalizations–Median (IQR)**		0 (0–1)	0 (0–1)

Patient characteristics including demographic data, cause of the bronchiectasis, and investigative results. ABPA = allergic bronchopulmonary aspergillosis; COPD = chronic obstructive lung disease; A1AT = alpha 1 antitrypsin deficiency; NTM = nontuberculous mycobacteria; CFTR = cystic fibrosis transmembrane conductance regulator; BSI = bronchiectasis severity index, SD = standard deviation

**Table 2:** Details of the diagnostic work-up and guarantee level of the diagnosis.

Pat No.	Typical symptoms	nNO	HVMA	TEM	IF	Geno- typing	Guarantee level of the diagnosis
**1, f38**	X	X	X	(X)	0	X	definite
**2, f33**	X	X	X	X	0	X	definite
**3, f27**	X	X	X	X	0	0	definite
**4, f34**	X	X	X	(X)	X	X	definite
**5, m43**	X	X	X	X	0	X	definite
**6, m51**	X	X	X	(X)	0	X	definite
**7, f32**	X	(X)	X	X	0	0	definite
**8, f44**	X	X	0	0	0	0	probable
**9, f20**	X	X	X	(X)	0	X	definite
**10, f31**	X	X	0	0	0	0	probable
**11, f22**	X	X	X	X	(X)	0	definite
**12, f35**	X	X	X	X	0	X	definite
**13, m40**	X	X	0	0	0	0	probable
**14, m19**	X	X	X	X	0	0	definite
**15, f38**	X	X	X	X	0	X	definite
**16, f39**	X	X	0	0	0	0	probable
**17, m19**	X	X	(X)	0	0	0	probable
**18, f27**	X	X	0	0	0	0	probable
**19, f37**	X	X	X	0	0	0	definite
**20, f25**	X	X	X	X	0	0	definite
**21, f24**	X	X	X	(X)	(X)	0	definite
**22, f42**	X	X	X	(X)	(X)	0	definite
**23, f60**	X	X	X	X	0	X	definite
**24, m43**	X	X	X	(X)	0	X	definite
**25, f43**	X	X	X	X	0	X	definite
**26, f41**	X	X	0	0	0	0	probable
**27, f20**	X	X	X	X	0	0	definite
**28, f56**	X	X	0	X	0	0	probable
**29, m50**	X	X	0	0	0	0	probable
**30, f72**	X	X	0	0	0	0	probable
**31, f47**	X	X	X	0	0	0	definite
**32, m41**	X	X	X	(X)	0	0	definite
**33, f59**	X	X	(X)	0	0	(X)	probable
**34, f19**	X	X	X	(X)	0	0	definite
**35, m32**	X	X	0	0	0	0	probable
**36, f59**	X	X	X	(X)	0	0	definite
**37, f51**	X	X	X	(X)	0	0	definite
**38, f33**	X	X	X	X	0	0	definite
**39, f67**	X	X	(X)	X	0	X	definite
**40, m73**	X	X	X	0	0	0	definite
**41, m62**	X	X	(X)	(X)	0	X	probable
**42, f43**	X	X	0	0	0	0	probable
**43, m75**	X	X	X	0	0	(X)	definite
**44, f32**	X	X	X	(X)	0	X	definite
**45, m29**	X	X	0	0	0	0	probable
**46, m29**	X	X	0	0	0	0	probable

Patients with PCD, bronchiectasis, and characteristic symptoms underwent clinical investigation, and the guarantee level of the diagnosis was according to Werner et al. [24]; X = investigation done with a positive result; (x) = investigation done with a negative result; 0 = investigation not done.

### CT features

In patients with PCD, the extent and severity of the bronchiectasis were significantly lower in the upper lung lobes (p<0.001) and higher in the middle and lower lobes compared to those of patients with bronchiectasis caused by other underlying diseases. Bronchial wall thickening was significantly less in the upper lobes and higher in the middle lobe / lingula (p = <0.001–0.040). The results regarding the involvement, bronchial dilatation, and bronchial wall thickening, according to Reiff [18], are given in Table 3. The lobar distribution (Table 4) differed significantly between patients with and those without PCD, whereas bronchiectasis in patients with PCD was predominant in the middle and lower lobes (<0.001). Descriptive data regarding the type of bronchiectasis in the different lobes (Table 5) confirm the higher prevalence of bronchiectasis in the middle and lower lobes in patients with PCD. Bronchiectasis in the upper lobes are present in only 30% of the patients with PCD compared to 50% of the patients with other underlying disorders. In patients with PCD, bronchiectasis is more often of the varicose type than that seen in patients without PCD.

**Table 3:** Reiff score.

Feature		overall			PCD			nonPCD			group comparison
		n	mean	median	n	mean	median	n	mean	median	p-value
**Involvement**	**right upper lobe**	121	0.93	1.00	46	0.43	0.00	75	1.23	1.00	<0.001
	**middle lobe**	113	1.44	2.00	43	1.62	2.00	72	1.34	2.00	0.108
	**right lower lobe**	119	1.53	2.00	46	1.62	2.00	74	1.47	2.00	0.386
	**left upper lobe**	120	0.67	0.00	47	0.20	0.00	76	0.95	1.00	<0.001
	**lingula**	120	1.19	1.00	46	1.38	2.00	75	1.08	1.00	0.063
	**left lower lobe**	114	1.49	2.00	43	1.55	2.00	72	1.46	2.00	0.505
**Bronchial dilatation**	**right upper lobe**	121	0.97	1.00	47	0.57	0.00	76	1.21	1.00	<0.001
	**middle lobe**	107	1.59	2.00	39	1.84	2.00	69	1.45	1.00	0.066
	**right lower lobe**	119	1.58	1.00	46	1.64	2.00	74	1.54	1.00	0.559
	**left upper lobe**	121	0.61	0.00	42	0.17	0.00	72	0.88	1.00	<0.001
	**lingula**	119	1.34	1.00	45	1.43	1.50	75	1.29	1.00	0.413
	**left lower lobe**	113	1.65	2.00	42	1.66	2.00	72	1.64	2.00	0.953
**Bronchial wall thickening**	**right upper lobe**	120	0.46	0.00	47	0.28	0.00	75	0.57	0.00	0.011
	**middle lobe**	108	0.93	1.00	39	1.21	1.00	70	0.77	1.00	0.004
	**right lower lobe**	117	1.15	1.00	45	1.25	1.00	73	1.10	1.00	0.314
	**left upper lobe**	121	0.27	0.00	47	0.07	0.00	76	0.40	1.00	<0.001
	**lingula**	119	0.76	1.00	45	0.93	1.00	75	0.65	1.00	0.040
	**left lower lobe**	112	1.16	1.00	42	1.32	1.00	71	1.07	1.00	0.143

Results of the Reiff-Score for all patients with bronchiectasis and separately for those with and without PCD. Group comparisons between patients with and without PCD were performed using the Mann-Whitney-U-test. Not all features could be evaluated in all lobes due to lobe resections and atelectasis.

**Table 4:** Lobar distribution.

	Overall (n = 121)	PCD (n = 46)	non PCD (n = 75)	Group comparison p-Value
**0 (widespread)**	23 (19%)	2 (4%)	21 (28%)	0.001
**1 (predominantly upper)**	7 (6%)	0 (0%)	7 (9%)	(0.033)*
**2 (predominantly middle)**	17 (14%)	9 (20%)	8 (11%)	0.172
**3 (predominently lower)**	69 (57%)	31 (67%)	38 (51%)	0.071
**4 (equally middle and lower)**	5 (4%)	4 (9%)	1 (1%)	(0.048)*
**predominantly middle and lower lobes**	91 (75%)	44 (96%)	47 (63%)	<0.001

Results of the lobar distribution of bronchiectasis, according to Reiff, for all patients with bronchiectasis and separately for those with and without PCD. Group comparisons between patients with and without PCD were performed using the Chi-square-test.

*Requirements for the Chi-square test are not fulfilled

**Table 5:** Type of bronchiectasis.

Lobe	Type of bronchiectasis	Overall (n = 121)	PCD (n = 46)	non PCD (n = 75)
**right upper lobe**	none	52 (43%)	32 (70%)	20 (27%)
	cylindrical	35 (29%)	6 (13%)	29 (39%)
	varicose	27 (22%)	8 (17%)	19 (25%)
	cystic	7 (6%)	0	7 (9%)
	resection	0	0	0
**middle lobe**	none	19 (16%)	3 (7%)	16 (21%)
	cylindrical	38 (31%)	11 (24%)	27 (36%)
	varicose	47 (39%)	25 (54%)	22 (29%)
	cystic	8 (7%)	2 (4%)	6 (8%)
	resection	9 (7%)	5 (11%)	4 (5%)
**right lower lobe**	none	15 (12%)	3 (7%)	12 (16%)
	cylindrical	48 (40%)	18 (39%)	30 (40%)
	varicose	45 (37%)	21 (46%)	24 (32%)
	cystic	11 (9%)	3 (7%)	8 (11%)
	resection	2 (2%)	1 (%)	1 (1%)
**left upper lobe**	none	76 (63%)	40 (87%)	36 (48%)
	cylindrical	22 (18%)	2 (4%)	20 (27%)
	varicose	17 (14%)	4 (9%)	13 (17%)
	cystic	6 (5%)	0	6 (8%)
	resection	0	0	0
**Lingula**	none	30 (25%)	7 (15%)	23 (31%)
	cylindrical	39 (32%)	12 (26%)	27 (36%)
	varicose	42 (35%)	22 (48%)	20 (27%)
	cystic	8 (7%)	3 (7%)	5 (7%)
	resection	2 (2%)	2 (4%)	0
**left lower lobe**	none	16 (13%)	5 (11%)	11 (15%)
	cylindrical	42 (35%)	15 (33%)	27 (36%)
	varicose	43 (36%)	17 (37%)	26 (35%)
	cystic	12 (10%)	4 (9%)	8 (11%)
	resection	8 (7%)	5 (11%)	3 (4%)

Results of the assessment of the type of bronchiectasis in the different lobes according to Reiff. Descriptive data are given for all patients with bronchiectasis and separately for those with and without PCD.

**Fig 1:**
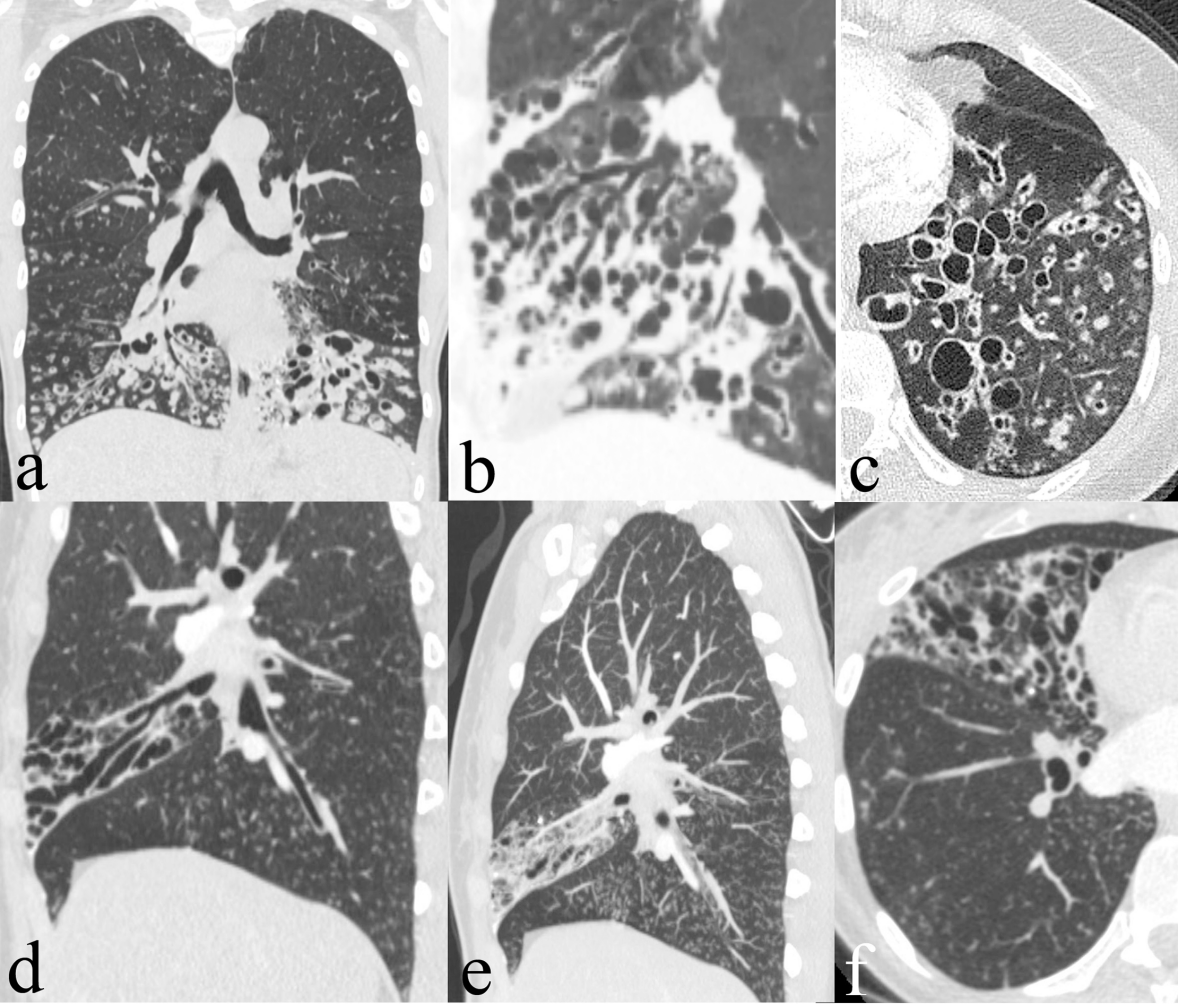
CT in patients with PCD. CT of two patients with definite PCD. Patient 1 (a-c) is a 34 year old female patient with PCD. CT shows cystic bronchiectasis especially in the lower lobes, severe mucous-plugging and tree in bud pattern. Patient 2 (d-f) is a 33 year old female patient. CT shows varicose bronchiectasis solely in the middle lobe, mucous-plugging and associated tree in bud pattern in the right lower lobe.

**Fig 2:**
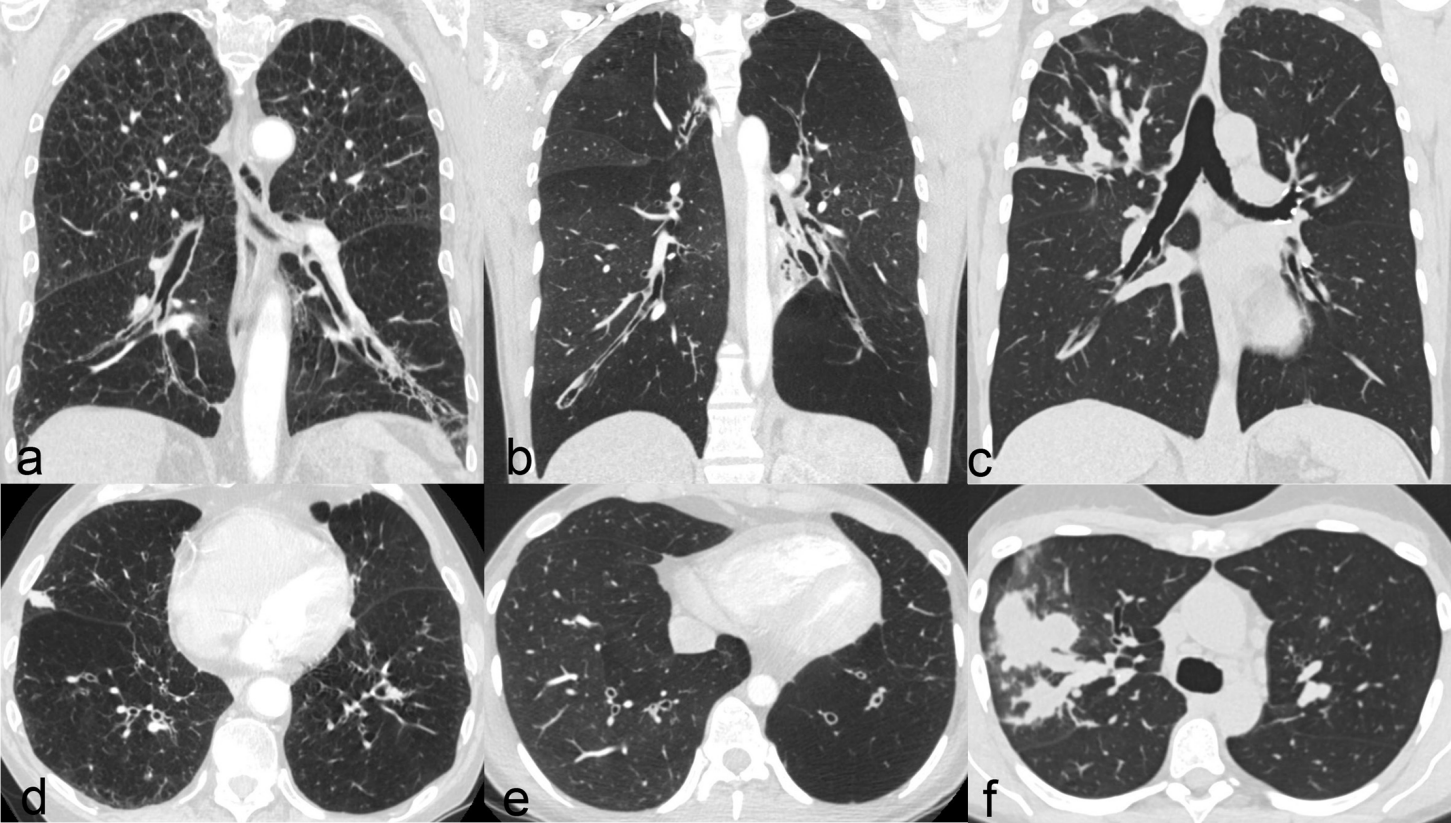
CT in patients with other underlying diseases. CT of three patients with bronchiectasis due to other underlying diseases. Patient 1 is a 74 year old male patient with COPD (a, d). Bronchiectasis are of cylindrical configuration and predominant in the lower lobes, collateral findings are the emphysematous changes. Patient 2 is a 20 year old woman with Swyer James Syndrome (b, e). There are severe bronchiectasis especially in the left lower lobe; the corresponding lung lobes are hyperinflated and the accompanying vessels are thin. Patient 3 (c, f) is a 53 year old female patient with allergic bronchopulmonary aspergillosis. There are focal varicose bronchiectasis in the right lower lobe with severe mucoid impactions.

Regarding collateral findings (Tables 6 and 7), inflammatory changes such as central (p = 0.050) and peripheral (p = 0.019) consolidations as well as central (p = 0.026) and peripheral (p = 0.013) ground glass opacities were significantly less common in patients with PCD than in those without PCD. Similarly, interstitial changes, such as emphysema (p = 0.003), interlobular thickening (p<0.001), and intralobular lines (p <0.001), were less frequent in patients with PCD compared to those with other diseases. Occurring significantly more often are mucous plugging (p = 0.001), a tree in bud pattern (p <0.001), and atelectasis (p = 0.009) in patients with PCD. Later, atelectasis of a total middle or lower lobe (p = 0.031), a history of resection of a middle or lower lobe (p = 0.047), and a predominance of bronchiectasis in the middle and lower lobes (p<0.001) are more frequent in patients with PCD. A situs inversus (Kartagener’s Syndrome) was only seen in patients with PCD (17%, p <0.001). To illustrate these findings, CT images of two patients with PCD are given in Fig 1 and of further three patients with bronchiectasis due to other underlying diseases in Fig 2.

**Table 6:** Collateral findings.

	Overall (n = 121)		PCD (n = 46)		non PCD (n = 75)		group comparison p-value
	mean	median	mean	median	mean	median	
**Mucus plugging**	1.17	1.00	1.46	2.00	0.99	1.00	0.001
**Tree in bud**	0.99	1.00	1.48	2.00	0.69	1.00	<0.001
**Consolidations peripheral**	0.46	0.00	0.26	0.00	0.59	0.00	0.019
**Consolidations central**	0.05	0.00	0.00	0.00	0.08	0.00	0.050
**Ground glass peripheral**	0.77	1.00	0.57	1.00	0.89	1.00	0.013
**Ground glass central**	0.20	0.00	0.07	0.00	0.28	0.00	0.026
**Interlobular thickening**	0.63	1.00	0.35	0.00	0.80	1.00	<0.001
**Intralobular lines**	0.58	0.00	0.20	0.00	0.81	1.00	<0.001

Results of the collateral findings in CT with an ordinary scale for all patients with bronchiectasis and separately for those with and without PCD. Mean values are given for the total patient group and separately for patients with and without PCD. Group comparisons were performed using the Mann-Whitney-U-test.

**Table 7:** Collateral findings.

	overall (n = 121)	PCD (n = 46)	non PCD (n = 75)	group comparison p-value
**Situs inversus**	8 (7%)	8 (17%)	0 (0%)	<0.001
**Emphysema**	21 (17%)	2 (4%)	19 (25%)	0.003
**Mosaic attenuation**	40 (33%)	14 (30%)	26 (35%)	0.631
**Atelectasis**	83 (69%)	38 (83%)	45 (60%)	0.009
**Cavity**	8 (7%)	1 (2%)	7 (9%)	0.124
**Atelectasis of a middle or lower lobe**	14 (12%)	9 (20%)	5 (7%)	0.031
**Volume loss in a middle or lower lobe**	42 (35%)	24 (52%)	18 (24%)	0.002
**History of resection of a middle / lower lobe**	21 (17%)	12 (26%)	9 (12%)	0.047

Results of collateral findings on CT with a binary scale for all patients with bronchiectasis and separately for those with and without PCD. Descriptive data are given for the total patient group and separately for patients with and without PCD. Group comparisons were performed using the Chi-square-test.

## Discussion

CT patterns in patients with PCD differ significantly from those in patients with bronchiectasis due to other underlying diseases. There was a greater prevalence of bronchiectasis in the middle and lower lobes, tree in bud pattern, atelectasis, and situs inversus in patients with PCD. Patients with other underlying diseases more often showed a predominance of bronchiectasis in the upper lobes as well as fibrotic and emphysematous changes of lung parenchyma.

Even though CT has a very high sensitivity and specificity for the diagnosis of bronchiectasis, in several, previously published studies it has been shown to be of limited value for identifying the underlying disease. Reiff et al. compared the special features of bronchiectasis in patients with different underlying diseases and could demonstrate differences in some CT features, although these features were of limited value for distinguishing diseases [18]. Lee et al. reported based on a study in which radiologists stated the most probable underlying disease in patients with bronchiectasis. A correct first-choice diagnosis was made in only 45% of the readings [21]. Cartier et al. obtained the correct diagnosis in 61% in 82 patients with a specific diagnosis of bronchiectasis and with the highest accuracy in CF (68%) and previous tuberculosis (67%) [22]. Even though in none of these studies could CT make the correct diagnosis in more than 2/3 of the patients, further studies regarding patients with PCD seems advantageous due to the particular findings that have been described in the context of PCD and the technical advances that have been made in CT imaging during the last decade.

Previous studies regarding PCD seen on CT revealed the predominance of bronchiectasis in the middle and lower lobes of the lungs [23]. Later, primarily varicose bronchiectasis, a chronic volume loss, a tree in bud pattern, and mucous plugging have been noted as having been seen [4]. Our results comply with this and revealed the prevalence of bronchiectasis in the lower or middle lobe / lingula in 89% of patients with PCD. A situs inversus was present 17% of the patients with PCD in our study cohort. This is less than the previously reported values of approximately 50% in patients with PCD [23] and demonstrates the late diagnosis of PCD in the adult patient cohort. As an atelectasis or a volume loss in the middle and lower lobes was also present in our study cohort in 19% / 51% of patients with PCD, the results were significantly higher than those in patients without PCD (p = 0.033 / p = 0.002). In addition to these previously described patterns in patients with PCD, we found a history of resection of a lower or middle lobe / lingula in more patients with PCD (26%) than in patients with non-PCD bronchiectasis (12%, p = 0.050). These may be specific findings for our adult bronchiectasis patient population with previously undiagnosed PCD. Furthermore, we found a significantly lower number of patients with intra- and interlobular lines in terms of fibrotic changes (p<0.001) or emphysema (p = 0.003) in the PCD subgroup. As far as we know, this has not been previously reported. Nevertheless, this result is not unexpected as other causes of bronchiectasis include COPD, emphysema, and traction bronchiectasis in patients with fibrosis. In contrast to children with PCD [26], in our study population all of the adult patients with PCD show bronchiectasis on CT and some of them had a history of resection of a pulmonary lobe. This demonstrates the severity of the disease with progression over years and the importance of an earlier diagnosis, dedicated management, and targeted treatment. Unfortunately some patients had a lobectomy before the diagnosis of PCD had been made. This increases the presumption that the disease is under-recognized. For the surety level of PCD, we used the criteria of the international registry for PCD from Werner et al. [24]. In 2017, the EU task force published guidelines for the diagnosis of PCD using another algorithm which proposes a set of quality criteria for future research and helps to assure this complex diagnosis [27]. The awareness of PCD is low among clinical practitioners, and the technical equipment required for the diagnostic work-up is available only in specialized medical centers. In contrast, nearly every adult patient with bronchiectasis and every patient in a registry for bronchiectasis has undergone a CT examination and particular findings for patients with PCD have been described [4,9,23]. Therefore, the potential of CT as a diagnostic test for identifying a patient’s underlying condition is currently underused and ours is the first study which shows that CT might be helpful, in addition to a patient’s medical history, for identifying patients in the bronchiectasis cohort in which further work-up for PCD is warranted.

The major limitation of our study is the small number of patients who have undergone CT in PCD. Therefore, further studies with more patients will be required. Nevertheless, PCD is a rare disease and this is the largest study addressing the use of CT for guiding the diagnosis in addition to a patient’s medical history and clinical features to date. Another limitation of our study is the use of different CT scanners and different CT protocols. However, we screened the studies for image quality and excluded studies that did not adhere to quality criteria set forth by the American Association of Physicists in Medicine [28].

## Conclusions

In conclusion, CT imaging features of bronchiectasis differ significantly in adult patients with PCD and in those with other underlying diseases. All of the adult patients with PCD had bronchiectasis, which underlines the severity of the disease and the importance of an earlier diagnosis and targeted treatment. Typical imaging features for PCD are a predominance of bronchiectasis in the middle and lower lobes, severe tree in bud pattern, atelectasis, and the absence of fibrotic and emphysematous changes. These findings may help clinical practitioners to identify patients with bronchiectasis in whom further complex work-up for PCD is warranted.

## Supporting information

S1 Table Results of CT evaluation. Data of CT evaluation including results of the Reiff score and collateral findings in patients with (clin_PCD = 1) and without (clin_PCD = 0) PCD.
(XLSX)
